# Unfolded protein response in bronchopulmonary dysplasia: mechanisms, pathways, and therapeutic implications

**DOI:** 10.3389/fcell.2026.1700811

**Published:** 2026-04-23

**Authors:** Haiyue Yu, Yongjing Guo, Xin Wang, Wanxu Guo, Yunfeng Zhang

**Affiliations:** 1 Department of Pediatrics, The Second Hospital of Jilin University, Changchun, China; 2 Department of Critical Care Medicine, The Second Hospital of Jilin University, Changchun, China; 3 Department of Neonatology, The Second Hospital of Jilin University, Changchun, China; 4 Children Disease Diagnosis and Treatment Center, The Second Hospital of Jilin University, Changchun, China

**Keywords:** bronchopulmonary dysplasia, endoplasmic reticulum stress, hyperoxia, lung development, neonatal lung injury, oxidative stress, unfolded protein response

## Abstract

Bronchopulmonary dysplasia (BPD) is a prevalent chronic lung disease in preterm infants, characterized by impaired alveolarization and abnormal vascular development. Growing evidence indicates that endoplasmic reticulum stress (ERS) and the unfolded protein response (UPR) are closely involved in the pathogenesis of BPD. Under hyperoxic conditions, excessive reactive oxygen species promote protein misfolding and accumulation within the endoplasmic reticulum, leading to activation of the three major UPR branches—IRE1, PERK, and ATF6. Dysregulation of these pathways contributes to lung injury and disrupted development through multiple stress-responsive cellular processes. This review summarizes current understanding of the molecular mechanisms by which UPR signaling participates in hyperoxia-induced lung injury in BPD and discusses therapeutic strategies with potential relevance to UPR modulation, including caffeine, vitamin A, and tauroursodeoxycholic acid (TUDCA), among others, based on available clinical and preclinical evidence. Although some agents have demonstrated clinical benefits in reducing BPD-related outcomes, whether these effects are mediated directly through UPR modulation remains uncertain. In contrast, several UPR-targeting compounds have shown promise in experimental models but lack clinical validation regarding safety and efficacy. Further studies are needed to clarify the cell-type-specific roles of UPR signaling in the developing lung, to better define the transition from adaptive to maladaptive UPR activation, and to support translational efforts that integrate mechanistic insights with clinical investigation. Such advances may help bridge the gap between molecular understanding and therapeutic development for BPD.

## 1 Introduction

Bronchopulmonary dysplasia (BPD) represents a major chronic condition linked to preterm birth (Shah et al., 2016). Despite global advancements in the management of premature infants, the incidence of BPD continues to rise annually (Nakashima et al., 2021; Zhicheng Zhu et al., 2023). The global incidence of BPD among extremely preterm infants (gestational age <28 weeks) varies widely, ranging from 17% to 75%, due to differences in gestational age, birth weight, disease definitions, treatment protocols, and survival rates across various study populations and institutions (Siffel et al., 2019). BPD negatively impacts the outcomes for preterm infants, resulting in higher rates of rehospitalization, neurodevelopmental issues, and pulmonary hypertension, which places a significant strain on both families and society (Kato et al., 2022).

The endoplasmic reticulum (ER) is an essential organelle within eukaryotic cells, playing a pivotal role in the folding and post-translational modification of secretory and membrane proteins. The efficiency and accuracy of protein folding are critical for maintaining ER homeostasis. Accumulation of a substantial number of unfolded and misfolded proteins within the ER lumen leads to a condition known as ER stress (ERS) (Venkatesan et al., 2023). To alleviate this stress and reestablish normal ER function, cells activate three primary signaling pathways: inositol-requiring enzyme 1 (IRE1), protein kinase RNA-like ER kinase (PERK), and activating transcription factor 6 (ATF6) (Rimaljot Singh et al., 2023), collectively known as the unfolded protein response (UPR).

UPR is intricately associated with various cellular physiological functions and is significantly implicated in the onset and progression of numerous diseases. Recent studies have substantiated the pivotal role of UPR in the pathophysiology of a wide array of diseases like tumors (Salvagno et al., 2022), liver and cardiovascular diseases (Ajoolabady et al., 2023; Chen and Zhang, 2023), neurological disorders (Wang et al., 2022), and ovarian diseases (Koike et al., 2023). Researchers are increasingly examining the link between BPD and ER stress, though the mechanisms are not yet fully understood. This review highlights the regulatory mechanisms linking the three branches of the unfolded protein response (UPR) to bronchopulmonary dysplasia (BPD) and provides a summary of recent pharmacological intervention strategies that target UPR pathways in the context of BPD. Additionally, we explore the potential connections between UPR and both the pathogenesis and therapeutic strategies of BPD.

## 2 Structure and function of the ER

Eukaryotic cells compartmentalize distinct functional regions through membranes, creating diverse environments for cellular processes (Li et al., 2023), with the ER being a crucial component of these compartments. Structurally, ER typically comprises stacked membrane sheets, forming a continuous membrane system that includes the nuclear envelope and the peripheral ER. The peripheral ER itself consists of both sheets and a polygonal tubular network (Terasaki et al., 2013). ER is the main site for protein folding and secretion, calcium storage, and lipid synthesis (Yu et al., 2020).

ER is characterized by a high redox potential and calcium concentration, akin to extracellular environments, rendering it conducive to the folding of secretory proteins (Bagur and Hajnoczky, 2017; Hetz and Papa, 2018). It is estimated that approximately one-third of proteins are directed towards folding within the ER (Pozzer et al., 2021). Following their synthesis on ribosomes, secretory and membrane proteins translocate into the ER lumen (Oakes and Papa, 2015), where they undergo a series of post-translational modifications. These modifications include the removal of signal peptides, the formation of disulfide bonds, and glycosylation. Proteins achieve their correct conformation with the aid of molecular chaperones and foldases before being dispatched to their respective functional sites (Hetz, 2020). Nevertheless, the attainment of correct protein conformations presents significant challenges, often resulting in protein misfolding. As a result, the ER not only acts as a central hub for protein production but also serves as a critical quality control organelle for synthesized proteins (Chen X. et al., 2023). The protein quality control system within the ER is essential for maintaining protein stability and includes mechanisms such as the unfolded protein response (UPR), ER-associated degradation (ERAD), and ER-related autophagy (ER-phagy) (Chen G. et al., 2023). These pathways form a complex signaling network to remove defective proteins, reduce ERS, and maintain ER protein homeostasis.

## 3 UPR signaling pathways

ER homeostasis is continually challenged by physiological demands and pathological insults. An elevation in protein secretion or disturbances in protein folding can result in the accumulation of unfolded or misfolded proteins within the ER lumen, leading to ERS (Hetz, 2020). The activation of UPR signaling pathways plays a pivotal role in mitigating ERS and preserving normal ER function.

**FIGURE 1:**
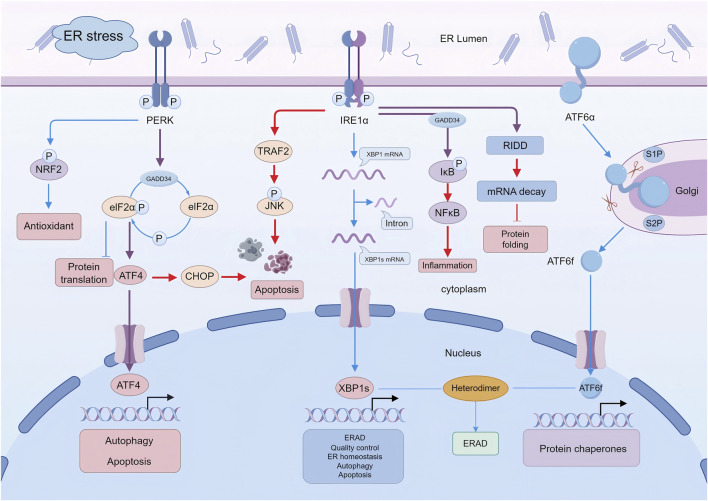
UPR signaling pathways. The unfolded protein response (UPR) is a multifaceted signaling network that is activated by endoplasmic reticulum (ER) stress to maintain proteostasis and cellular homeostasis. Upon ER stress, the three principal UPR sensors–PERK, IRE1α, and ATF6α–initiate distinct yet interconnected signaling cascades that regulate protein translation, folding, degradation, and stress-adaptive gene expression. Depending on the intensity and duration of ER stress, UPR signaling can promote adaptive responses aimed at restoring ER homeostasis or shift toward maladaptive outputs that contribute to cellular dysfunction, inflammation, and apoptosis. In the schematic, blue arrows indicate predominantly adaptive or homeostatic pathways, red arrows denote pro-inflammatory or pro-apoptotic signaling associated with severe or prolonged ER stress, and purple arrows highlight context-dependent regulatory nodes with dual roles. ER, endoplasmic reticulum; PERK, PKR-like ER kinase; IRE1, inositol-requiring enzyme 1; ATF6, activating transcription factor 6; NRF2, nuclear factor erythroid 2-related factor 2; GADD34, growth arrest and DNA damage-inducible protein 34; eIF2, eukaryotic translation initiation factor 2; ATF4, activating transcription factor 4; CHOP, C/EBP homologous protein; TRAF2, tumor necrosis factor receptor–associated factor 2; JNK, c-Jun N-terminal kinase; XBP1, X-box binding protein 1; XBP1s, spliced XBP1; NF-κB, nuclear factor κB; ERAD, ER-associated protein degradation; RIDD, regulated IRE1-dependent decay; ATF6f, N-terminal fragment of ATF6.

These UPR signaling pathways are initiated through three ER-resident transmembrane proteins: IRE1, PERK, and ATF6 (Izadpanah et al., 2023). While two primary hypotheses exist regarding the activation of UPR due to the accumulation of unfolded proteins in the ER lumen—namely, the direct binding and indirect recognition models—some researchers propose that these models may be complementary (Vitale et al., 2019). Irrespective of the underlying mechanism, the binding immunoglobulin protein (BiP), also referred to as glucose-regulated protein 78 (GRP78), is recognized as playing a pivotal role in the regulation of UPR, a fact established for more than 2 decades (Bertolotti et al., 2000). BiP functions as an ER-resident molecular chaperone and is notably abundant in mammalian cells, comprising approximately 5% of the ER lumen’s contents (Gething and Sambrook, 1992). Under typical physiological conditions, BiP binds to three transmembrane proteins to suppress the activation of the UPR. In contrast, during ERS, BiP dissociates from these proteins due to its preferential binding to misfolded or unfolded proteins, thereby triggering UPR activation (She et al., 2023). Upon activation, UPR pathways initiate downstream signaling cascades that serve to attenuate protein translation, augment protein folding capacity, and re-establish ER homeostasis. As a multifaceted signaling network, the UPR initiates adaptive responses through various signaling pathways to restore ER homeostasis (Figure 1).

### 3.1 UPR pathway proteins

#### 3.1.1 IRE1

Among these pathways, IRE1 is recognized as the most evolutionarily conserved UPR pathway in metazoans. Mammals possess two homologs of IRE1 (Tirasophon et al., 1998; Wang et al., 1998), namely, IRE1α and IRE1β. IRE1α is ubiquitously expressed across various tissues, whereas IRE1β expression is specific in mucosal epithelial cells, such as those found in the respiratory and gastrointestinal tracts. Extensive research has been conducted on IRE1α, in contrast to the limited understanding of IRE1β’s function (Cloots et al., 2021). Therefore, references to the IRE1 pathway typically pertain to IRE1α.

IRE1α is characterized as a bifunctional transmembrane protein, featuring an ER luminal domain, a transmembrane region, and a cytoplasmic domain that encompasses serine/threonine kinase and RNA endonuclease activities. Under ERS, IRE1α undergoes dimerization and autophosphorylation of its adjacent kinase domains, which subsequently activates its site-specific RNA endonuclease activity (Na et al., 2023). The activated RNA endonuclease specifically recognizes and splices X-box binding protein 1 (XBP1) mRNA, excising a 26-nucleotide intron to generate the transcription factor XBP1 (Cox et al., 1993; Mori and Ma, 1993; Tirasophon et al., 1998). Then XBP1 translocates to the nucleus, where it upregulates genes associated with survival mechanisms, including quality control, ER homeostasis, autophagy, and apoptosis, thereby facilitating the cellular response to ERS (Divya and Ravanan, 2023). In the absence of XBP1, IRE1α initiates regulated IRE1-dependent decay (RIDD) (Abdullah and Ravanan, 2018; Tang et al., 2018), resulting in the degradation of specific mRNAs and microRNA precursors, thereby influencing cellular functions and alleviating the protein-folding load in the ER (Maurel et al., 2014; Ottens et al., 2023). The escalation of RIDD activity is directly associated with the intensity and duration of ERS, while the splicing of XBP1 mRNA remains unaffected (Hollien and Weissman, 2006). During prolonged ERS, IRE1α binds to tumor necrosis factor receptor-associated factor 2 (TRAF2), which enhances the phosphorylation of c-Jun N-terminal kinase (JNK), potentially leading to apoptosis (Urano et al., 2000) or the formation of a complex with cytoplasmic IκB kinase (IKK), phosphorylating IκB, and activating NF-κB to stimulate the production of inflammatory cytokines (Lei et al., 2023).

#### 3.1.2 PERK

PERK, similar to IRE1α, is a type I transmembrane protein kinase that becomes activated through dimerization, trans-autophosphorylation, and subsequent oligomerization (Divya and Ravanan, 2023). Once activated, PERK phosphorylates the α-subunit of the eukaryotic translation initiation factor 2 (eIF2α), thereby inhibiting the regeneration of the active eIF2 complex (Zhang et al., 2021). This action results in a global reduction in translation and a concomitant inhibition of protein synthesis (Harding et al., 1999; Yan et al., 2023), which serves to mitigate the protein-folding load within the ER. Notably, phosphorylated eIF2α also facilitates the translation of mRNAs containing upstream open reading frames, thereby promoting selective translation of transcription factor 4 (ATF4) (Vattem and Wek, 2004). ATF4 upregulates a variety of associated genes, including DNA damage-inducible gene 34 (GADD34) and the apoptosis signaling molecule C/EBP-homologous protein (CHOP), thereby modulating apoptosis and autophagy (Gong et al., 2017). Furthermore, the upregulation of ATF4 expression can influence the expression of BiP, which counteracts CHOP induction and promotes cell survival (Lan et al., 2023). GADD34 can also exert negative feedback to inhibit eIF2α phosphorylation. Additionally, PERK phosphorylates nuclear factor erythroid 2-related factor 2 (NRF2) (Woo et al., 2024), a transcription factor that serves as a key activator of antioxidant enzymes such as superoxide dismutase-1 (SOD1), HO-1, and CAT, thereby mitigating oxidative stress (Abu-Elfotuh et al., 2023). However, under conditions of severe ERS, activating transcription factor 4 (ATF4) can suppress the expression of the anti-apoptotic B-cell lymphoma 2 (Bcl2) protein via the C/EBP homologous protein (CHOP) pathway. This suppression facilitates the expression of Bax, ultimately leading to apoptosis (Hu et al., 2018). Above all, the activation of PERK generally serves to reduce the protein load within the ER, but failure of these mechanisms to restore ER homeostasis results in the initiation of cell death via the PERK pathway.

#### 3.1.3 ATF6

Unlike the transmembrane proteins mentioned above, ATF6 is a type II transmembrane protein that exists in two isoforms: ATF6α and ATF6β (Divya and Ravanan, 2023). Mice with individual knockouts of either isoform are viable, but the simultaneous knockout of both isoforms results in early embryonic death (Yamamoto et al., 2007). ATF6β may function as a transcriptional repressor, counteracting the activity of ATF6α to safeguard cells from ERS, although its roles may vary across different tissues (Ye, 2020). During ERS, ATF6α is translocated from the ER to the Golgi apparatus via vesicular transport, where it undergoes proteolytic cleavage by the proteases S1P and S2P. This cleavage releases the N-terminal fragment (ATF6f), which subsequently translocates to the nucleus. In the nucleus, ATF6f acts as a transcription factor, promoting the expression of genes that encode protein chaperones such as BiP, ER protein 57 (ERp57), GRP94, and ERAD (Wang and Kaufman, 2016). These actions collectively enhance the protein-folding capacity of the ER and contribute to the restoration of ER homeostasis. ATF6f is also capable of activating CHOP and enhancing the transcription of XBP1 (Cui X. et al., 2022; D’Amico et al., 2022). Furthermore, ATF6f can form heterodimers with spliced XBP1, thereby facilitating the expression of genes associated with ERAD (Yamamoto et al., 2007).

### 3.2 UPR and other cellular processes

In response to prolonged ERS, the UPR can trigger pathways involving inflammation, ERAD, autophagy, and apoptosis to re-establish ER homeostasis. Nevertheless, excessive ERS can result in a terminal UPR, which may lead to apoptosis and subsequent tissue or organ damage (Divya and Ravanan, 2023).

#### 3.2.1 Inflammation

**FIGURE 2:**
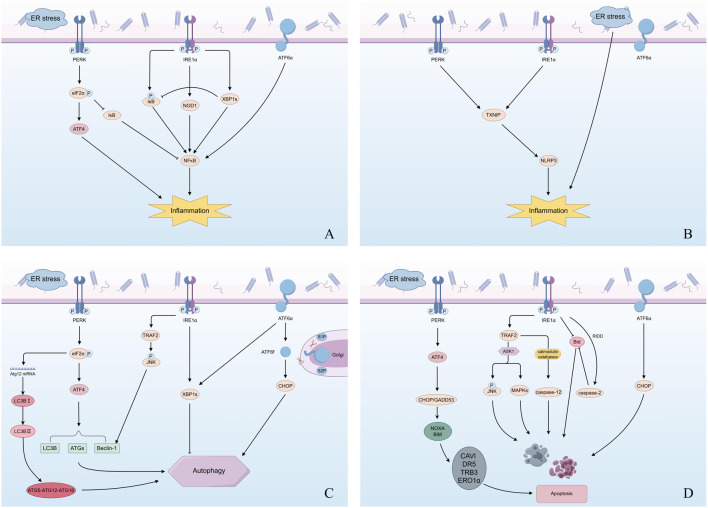
UPR and other cellular processes during prolonged ER stress. **(A)** UPR - NF-κB inflammatory axis. Persistent ER stress activates the three UPR branches PERK, IRE1α, and ATF6α. PERK phosphorylates eIF2α, which not only induces ATF4 upstream but also suppresses IκB translation, thereby releasing NF-κB inhibition. IRE1α promotes IκB phosphorylation, upregulates NOD1 and XBP1s, while XBP1s can inhibit phosphorylated IκB. Phosphorylated IκB, NOD1, and XBP1s together converge on NF-κB activation. The ATF6 pathway can also activate NF-κB. **(B)** UPR - NLRP3 inflammasome axis. PERK and IRE1α upregulate TXNIP, which subsequently activates the NLRP3 inflammasome; ATF6 may also participate in amplifying the inflammatory response. ER stress itself can enhance basal inflammatory signaling. **(C)** UPR - autophagy. When ERAD fails to eliminate misfolded proteins, autophagy is mobilized to restore homeostasis. PERK–eIF2α–ATF4 signaling upregulates Atg12 mRNA, facilitating LC3B-I to LC3B-II conversion and assembly of the ATG5–ATG12–ATG16 complex. ATF4 also transcriptionally induces LC3B, ATGs, and Beclin-1. IRE1α promotes autophagy via the TRAF2–JNK pathway as well as XBP1s. ATF6 is cleaved in the Golgi by S1P/S2P to generate ATF6f, which can indirectly regulate autophagy through CHOP and XBP1s. Autophagy initially plays an adaptive protective role, but excessive activation may shift cells from survival to death. **(D)** UPR - apoptosis. Under irreversible ER stress, PERK–ATF4 signaling upregulates CHOP/GADD153, which induces pro-apoptotic factors such as NOXA and Bim, as well as CHOP target genes including CAVI, DR5, TRB3, and ERO1α, driving apoptosis. IRE1α induces apoptosis via the TRAF2–ASK1–JNK/MAPK cascade and can also activate caspase-12 through calmodulin-dependent mechanisms. In addition, IRE1α-mediated RIDD activity together with Bid/caspase-2 signaling promotes apoptosis. ATF6 contributes by enhancing CHOP expression, thereby cooperating with the above pathways. ER, endoplasmic reticulum; PERK, PKR-like ER kinase; IRE1, inositol requiring enzyme 1; ATF6, activating transcription factor 6; eIF2, eukaryotic translation initiation factor 2; XBP1, X-box binding protein 1; XBP1s, spliced XBP1; NOD 1, nucleotide-binding oligomerization domain-containing protein 1; NF-κB, nuclear transcription factor κB; TXNIP,thioredoxin-interacting protein; NLRP3, NOD-,LRR-and pyrin domain-containing protein 3; ATGs, autophagy-related genes; LC3B, Microtubule-associated protein 1A/1B-light chain 3 beta; ATF4, activating transcription factor 4; JNK, c-Jun N-terminal kinase; CHOP, C/EBP-homologous protein; ATF6f, the N-terminal fragment of ATF6; CAVI, carbonic anhydrase VI; DR5, death receptor 5; TRB3, Tribbles homolog 3; ASK1, Recombinant Apoptosis Signal Regulating Kinase 1; MAPKs, mitogen-activated protein kinases; TRAF2, tumor necrosis factor receptor associated factor 2; RIDD, regulated IRE1-dependent decay.

Even in the absence of overt or insurmountable stimuli, the activation of UPR can induce the production of low levels of inflammatory cytokines (Yu et al., 2022). All three UPR proteins play a role in the pro-inflammatory process during ERS (Figures 2A, B). The nuclear transcription factor κB (NF-κB) serves as a pivotal regulator of inflammation and is integral to ERS-mediated inflammatory responses (Urano et al., 2000; Ying Fu et al., 2023). In addition to the IκB pathway previously discussed (Section 3.1.1), IRE1α can stimulate the production of nucleotide-binding oligomerization domain-containing protein 1 (NOD1) (Hu et al., 2023), which subsequently activates NF-κB. This activation initiates the expression of cytokine genes and the release of inflammatory mediators, thereby potentially linking ERS to inflammation (Keestra-Gounder et al., 2016). Furthermore, the IRE1/XBP1 pathway further amplifies NF-κB activation. The downregulation of XBP1 and the reduction of IRE1α phosphorylation lead to a decrease in the expression of inflammatory molecules. Conversely, the overexpression of XBP1 obstructs the IRE1α/IKK/NF-κB signaling pathway (Yu et al., 2015). Activation of PERK could promote the phosphorylation of eIF2α, inhibits the translation of IκB, and enhance NF-κB activity (Duan et al., 2022) and subsequently activate downstream inflammation-related genes such as TNF-α and interleukin-1 (IL-1) (Deng et al., 2004; Hotamisligil, 2010). Furthermore, ATF4 has been shown to induce the expression of inflammatory cytokines in human endothelial cells (Hotamisligil, 2010). Inhibition of BiP is associated with a reduction in inflammatory mediator levels and prevents the activation of NF-κB via the ATF6 pathway (Nakajima et al., 2011).

In addition to these mechanisms, ERS can exacerbate or initiate inflammation through the activation of the NOD-like receptor family pyrin domain-containing protein 3 (NLRP3) inflammasome, a critical element of the innate immune system. The assembly of the NLRP3 inflammasome leads to the activation of caspase-1, which subsequently induces the maturation and release of interleukin-1β (IL-1β) and interleukin-18 (IL-18), thereby contributing to the pathogenesis of inflammatory diseases (Schroder and Tschopp, 2010; Xu and Núñez, 2023). IRE1α and/or PERK can promote the upregulation of thioredoxin-interacting protein (TXNIP), which in turn activates the NLRP3 inflammasome (Lerner et al., 2012; Oakes, 2017). Furthermore, ERS has the capacity to directly activate the NLRP3 inflammasome, leading to increased IL-1β secretion independently of the UPR (Menu et al., 2012).

#### 3.2.2 ERAD

To eliminate misfolded proteins from the ER, the ER’s quality control system directs them towards ER-associated protein degradation (ERAD) via the proteasome (Kny and Fielitz, 2022). Traditionally, ERAD has been regarded as a component of the UPR, given that many of its mediators are regulated by UPR transcription factors (Nakada et al., 2021). Recent studies propose that ERAD functions as an independent protein quality control pathway that closely interacts with the UPR to maintain cellular protein homeostasis (Hwang and Qi, 2018). Investigations into UPR targets involved in ERAD demonstrate a high degree of coordination between the two systems: effective ERAD necessitates a functional UPR, and the induction of the UPR enhances ERAD capacity (Travers et al., 2000).

The fundamental process of ERAD encompasses substrate recognition, ubiquitination, retrotranslocation, and subsequent proteasomal degradation (Sun and Brodsky, 2019). Ubiquitination plays a pivotal role in facilitating the retrotranslocation of substrates. E3 ubiquitin ligases are integral to the ERAD pathway, as they mediate interactions between various ER luminal and cytoplasmic proteins. Misfolded proteins are translocated to the cytoplasm through the collaborative efforts of E3 ubiquitin ligases and associated proteins, ensuring efficient degradation (Qu et al., 2021). Regarding non-glycosylated substrates, BiP associates with these substrates and interacts with Derlin1 and the homocysteine-responsive endoplasmic reticulum-resident ubiquitin-like domain member 1 protein (HERP). HERP, which contains a ubiquitin domain, is likely involved in the retranslocation of substrates and their subsequent association with the proteasome as part of the Hrd1 complex (Li et al., 2020). The Hrd1 complex is mainly regulated by the IRE1α/XBP1 signaling pathway (Travers et al., 2000). Notably, IRE1, one of the activation sensors of the unfolded protein response (UPR), serves as a substrate for the Hrd1-ERAD complex (Sun et al., 2015). PERK is intricately associated with ERAD, and its inhibition interferes with protein trafficking from the ER to the Golgi apparatus, retrotranslocation from the ER to the cytoplasm, and subsequent proteasomal degradation (Gupta et al., 2010). Research suggests that eIF2α serves as a substrate for Hrd1, with Hrd1 facilitating its ubiquitination and proteasomal degradation (Yan et al., 2016; Huang et al., 2017). The involvement of ATF6 in ERAD has been previously discussed (see Section 3.1.3). Consequently, all three UPR pathways are integral to ERAD, thereby contributing to the maintenance of ER homeostasis.

#### 3.2.3 Autophagy

Originally characterized as a catabolic mechanism responsible for transporting damaged organelles, misfolded proteins, and various intracellular constituents to lysosomes for degradation (Dashti et al., 2024), autophagy encompasses three primary subtypes: macroautophagy, chaperone-mediated autophagy (CMA), and microautophagy (Mizushima et al., 2008). Typically, macroautophagy, often simply termed autophagy, serves as the principal pathway for sequestering cytoplasmic components destined for lysosomal degradation. In instances where ERS arises and the ERAD pathway is insufficient to alleviate it, autophagy is activated to facilitate the degradation of misfolded or unfolded proteins (Yang et al., 2024).

Autophagy is intricately linked to the activation of various UPR pathways (Figure 2C). It can be triggered by IRE1α through the activation of JNK or by the PERK/ATF4 axis under diverse stress conditions, leading to the upregulation of several autophagy-related genes (ATGs), Beclin-1, and microtubule-associated protein light chain 3 beta (LC3B) (Alhasan et al., 2023). The activation of the IRE1/TRAF2/JNK signaling pathway during ERS may regulate autophagy by influencing the function and expression of Beclin-1. Furthermore, the deletion of XBP1 in amyotrophic lateral sclerosis models has been shown to enhance autophagy and protect against the toxicity induced by aggregates of the enzyme superoxide dismutase 1, suggesting that the IRE1 pathway of the UPR may play a distinct role from TRAF2/JNK in regulating autophagy (Hetz et al., 2009; Verfaillie et al., 2010). Activation of the PERK/eIF2α pathway during ERS upregulates Atg12 mRNA, facilitating the conversion of LC3-I to LC3-II, which in turn activates the ATG5-ATG12-ATG16 complex and initiates autophagy (Kouroku et al., 2006). Furthermore, research indicates that the reduction of ERS through pharmacological intervention and the silencing of ATF4 lead to a downregulation of LC3B expression (Tyagi et al., 2023). Despite these findings, it is suggested that ATF4 and PERK may regulate autophagy via distinct mechanisms. Specifically, ATF4 is involved in transcriptional regulation and is crucial for the formation of autophagosomes, whereas PERK operates in a transcription-independent manner, playing a critical role in the post-sequestration stages of the autophagy pathway (Luhr et al., 2019). Direct evidence supporting the regulation of autophagy by ATF6 is predominantly observed in the context of viral infections, wherein the activation of ATF6 is essential for the induction of autophagy (Bahamondes Lorca and Wu, 2023). Typically, ATF6 is considered to regulate autophagy indirectly through the modulation of XBP1s and CHOP expression (Zhang S. et al., 2018).

In conclusion, during prolonged ERS, autophagy seems to serve as a final mechanism to reestablish ER homeostasis (Qi and Chen, 2019). Although autophagy generally plays a protective role, its excessive activation can ultimately transition cells from a survival state to a mode of cell death.

#### 3.2.4 Cellular senescence

Senescence represents a progressive condition of tissues initiated by endogenous stress and alterations in the microenvironment, such as metabolic, hormonal, and inflammatory changes, and is indicative of acute and adaptive responses (Salminen et al., 2020). This process is linked to conditions that are known to induce ERS, including autophagy and mitochondrial dysfunction, disturbances in calcium homeostasis, elevated oxidative stress, and disruptions in protein homeostasis and energy metabolism (Lopez-Otin et al., 2013). A characteristic feature of senescence is the reduction in protein synthesis (Tavernarakis, 2008), with the accumulation of senescent cells contributing significantly to tissue pathology (Deursen, 2014). Cellular senescence is a complex pathological and physiological phenomenon characterized by the irreversible cessation of cellular proliferation, while the cells remain metabolically active (Galluzzi et al., 2018). This state is associated with the extensive release of cytokines and immune-related molecules (Azizi et al., 2023), collectively known as the senescence-associated secretory phenotype (SASP) (Galluzzi et al., 2018).

The activation of the UPR is positively correlated with cellular senescence (Pluquet et al., 2015). Inducers of the UPR have been shown to elevate the expression of cyclooxygenase-2 (COX2) and lead to the excessive secretion of prostaglandin E2 (PGE2), thereby contributing to premature senescence. Conversely, the silencing of ATF6α and IRE1α has been found to inhibit the upregulation of COX2 and the production of PGE2 in replicative senescent fibroblasts (Cormenier et al., 2018). Further studies corroborate that the ERS/ATF6α axis is instrumental in mediating senescence induced by various stimuli, with the transcription factor activity of ATF6α being essential for the induction of senescence (Kim et al., 2018). IRE1α, through its RNase activity, is pivotal in the induction of the ischemic retinal cell senescence phenotype and the senescence-associated secretory phenotype (SASP) (Oubaha et al., 2016). The p53/p21WAF1 pathway, activated by accumulated DNA damage, constitutes the primary mechanism underlying senescence-related cell cycle arrest (Campisi, 2005). Evidence indicates that ERS-dependent activation of p21 signaling facilitates the promotion of induced cellular senescence (Liu et al., 2014). Numerous studies have shown that the upregulation of the ATF4 target gene CHOP/Gadd153 supports the involvement of the PERK pathway in cellular senescence (Pluquet et al., 2015). While there remains some debate regarding the causal relationship between UPR and cellular senescence, their close association is indisputable.

#### 3.2.5 Apoptosis

The term “apoptosis,” first introduced by Kerr in 1972, is derived from the Greek word describing the “falling off” of petals from flowers or leaves from trees (Kerr et al., 1972). Apoptosis, also known as “programmed cell death,” is a highly regulated process involving multiple sub-programs to ensure its proper initiation and execution (Mehrbod et al., 2019). Apoptosis can be initiated via the “extrinsic pathway,” which is characterized by the activation of membrane death receptors, or through the “intrinsic or mitochondrial pathway,” which involves increased mitochondrial outer membrane permeability (MOMP) and cytochrome c release (Keramidas et al., 2023).

In cases where ERS becomes irreversible, it frequently induces intrinsic apoptosis (Figure 2D). This process is regulated by the Bcl-2 family of proteins, including Bid, Bad, Bik, Buff, Bim, PUMA, NOXA, Box, Bok, Back, Bcl-2, and Bcl-xL (Cory and Adams, 2002; Czabotar and Garcia-Saez, 2023), which control the release of specific caspase activators within the mitochondria (Qu et al., 2021). Under conditions of abnormal and prolonged stress, the PERK pathway induces the expression of C/EBP homologous protein (CHOP/GADD153) via activating transcription factor 4 (ATF4), subsequently activating pro-apoptotic factors, like NOXA and Bim. The pro-apoptotic molecules activated by CHOP include carbonic anhydrase VI (CAVI), death receptor 5 (DR5), Tribbles homolog 3 (TRB3), and ERO1α, which collectively facilitate the apoptotic process (Beilankouhi et al., 2023). Additionally, ATF6 is involved in the regulation of CHOP expression, thereby contributing to the modulation of apoptosis (Fen Zhang et al., 2022). In contrast, IRE1α may decrease Bid levels through its RNase domain, leading to the degradation of pro-survival mRNA and microRNA via regulated IRE1-dependent decay (RIDD), and subsequently activating caspase-2-mediated Bid cleavage (Gsottberger et al., 2023). Bid facilitates cytochrome c release and subsequent apoptosis (Sahoo et al., 2023). Upon activation, IRE1α forms a trimeric complex with TRAF2, which subsequently activates JNK and p38 mitogen-activated protein kinases (MAPKs), further promoting apoptotic pathways (Yin et al., 2022; Zhao Li et al., 2023; Zhang R. et al., 2023). Moreover, the IRE1α-TRAF2 can activate caspase-12 via calmodulin catabases, leading to caspase-mediated apoptosis (Tomicic et al., 2015; Almanza et al., 2019), which can be mitigated by inhibiting the IRE1α/XBP1s/caspase-12 signaling cascade (Chang et al., 2021).

## 4 ERS in BPD

Bronchopulmonary dysplasia (BPD), also referred to as “chronic lung disease (CLD)” (Boel et al., 2022), is a chronic condition characterized by persistent respiratory complications and impaired lung function beginning in infancy (Everitt et al., 2021). BPD is characterized by lung damage arising from the interplay of various factors, including immature lung development, exposure to inflammatory mediators during fetal and neonatal stages, oxidative stress, and aberrant growth factor signaling. These interactions disrupt alveolar and vascular growth post-lung injury, leading to abnormal lung development patterns such as reduced alveolarization, interstitial thickening, and vascular disruption (Dankhara et al., 2023). While the precise pathogenesis of BPD remains complex and not entirely elucidated, accumulating evidence indicates that ERS is involved in BPD-associated airway inflammation, cellular senescence, autophagy, apoptosis, and post-inflammatory repair, playing a crucial role in BPD pathogenesis.

Hyperoxia represents the predominant risk factor for BPD and serves as a critical mediator of acute lung injury and infection (Amarelle et al., 2021; Gilfillan et al., 2021). The generation of reactive oxygen species (ROS) during hyperoxic conditions induces oxidative stress (Soundararajan et al., 2022), resulting in protein misfolding within ER and subsequently initiating ERS (Sidramagowda Patil et al., 2022).

### 4.1 Hyperoxia-induced UPR in BPD patients

Extremely or ultra-preterm infants frequently require mechanical ventilation and supplemental oxygen therapy, which are essential for enhancing survival rates. Nevertheless, the maturation of numerous antioxidant systems occurs concurrently with surfactant development, resulting in their underdevelopment in preterm infants. Consequently, these systems are insufficient to counteract the elevated ROS levels generated by hyperoxia ex utero, making them susceptible to oxidative stress (Valencia et al., 2018; Ganguly et al., 2021). Elevated ROS resulting from hyperoxia represent a significant contributor to oxidant-induced lung injury in BPD and corresponding animal models (Harijith et al., 2016). ROS are biomolecules characterized by the presence of at least one oxygen atom, exhibiting greater reactivity than molecular oxygen (O_2_). This category encompasses both non-radical and free radical species, the latter possessing at least one unpaired electron, such as hydrogen peroxide (H_2_O_2_), hydroxyl anion (OH-), and superoxide anion radical (O2-), which serve as essential redox signaling molecules (Resende et al., 2022). The main sources of ROS production include the mitochondrial electron transport chain (ETC) and the tricarboxylic acid cycle, with additional contributions from peroxisomes and the ER (Huang et al., 2021). Initially, hyperoxia impairs ETC, thereby diminishing mitochondrial oxygen consumption. This reduction exacerbates tissue hyperoxia and elevates the production of ROS within both mitochondrial and cytoplasmic compartments (Baik et al., 2023). ROS are integral to numerous biological processes, such as cell proliferation, immune response modulation, embryonic development, sperm capacitation, and the activation of transcription factors (Negri et al., 2021). Nevertheless, the overabundance of ROS results in oxidative stress, which can trigger apoptosis, inflict DNA damage, and compromise the functionality of proteins and lipids (Wang P. et al., 2023).

ROS directly target free thiol groups, which are crucial for the activity of protein-folding enzymes, resulting in oxidative modifications of ER luminal proteins. This process induces dysfunction in molecular chaperones and leads to the accumulation of unfolded proteins within the ER lumen, thereby initiating ERS (Cui X. et al., 2022). Subsequently, ERS activates UPR signaling pathway, which further elevates ROS production during both acute and chronic ERS conditions (Santos et al., 2009). Consequently, ROS instigate ERS, which in turn amplifies ROS levels (Lee and Song, 2021), establishing a self-perpetuating cycle that contributes to the pathogenesis of BPD through downstream molecular interactions.

### 4.2 Hyperoxia-induced UPR and other cellular processes in BPD

#### 4.2.1 Inflammation

In immature lungs, exposure to supraphysiological levels of oxygen and ROS triggers intricate inflammatory responses characterized by the accumulation of inflammatory cells, including neutrophils and alveolar macrophages, as well as cytokines. This cascade ultimately results in increased microvascular permeability and lung damage (Patel et al., 2010). As previously discussed, all three branches of UPR have the capacity to initiate inflammatory responses.

In animal models of hyperoxia-induced BPD, there is a marked elevation of pro-inflammatory cytokines such as IL-1β, IL-17, IL-6, and IFN-γ in the bronchoalveolar lavage fluid (BALF) (Wang J. et al., 2023). Similarly, increased levels of neutrophils and the pro-inflammatory cytokine IL-8 have been detected in the BALF of BPD patients (D’Angio et al., 2002). Research conducted by Petra Um-Bergström et al. demonstrates that the pattern of airway T cell subsets in young BPD patients (n = 22; median age 19.6 years) parallels that observed in patients with COPD, characterized by an elevated presence of cytotoxic T cells (Petra Um-Bergström et al., 2022). This finding implies that the lungs of BPD patients are in a state of inflammation. Importantly, the activation of UPR and the elevation of inflammatory mediators frequently occur simultaneously in animal models of hyperoxia-induced BPD, and the application of ERS inhibitors or blockers has been shown to mitigate inflammation and enhance alveolar development (Table 1).

**TABLE 1:** Changes of ERS-related markers and inflammation-related markers after hyperoxia.

Experimental model	ERS related	Inflammation related	References
Rat	p-PERK*, p-eIF2α*, ATF4*, CHOP*	TNF-α*, IL-6*, IL-1β*	Cui H. et al. (2022)
Mice	(2 days) BiP*, CHOP** (7 days) CHOP mRNA*, BiP*, IRE1α*, ATF6α*, PERK* CHOP*, XBP1 mRNA*	(2 days) COX mRNA* (7 days) COX mRNA*	Choo-Wing et al. (2013)
Rat	GADD153*, p-IRE1α/IRE1α*, p-JNK/JNK*	Total cell*, neutrophil*, macrophage*	Zhang S. et al. (2023)
Rat	BiP*, PERK*, IRE1*, ATF6**, p-PERK*, p-IRE1*, cATF6* CHOP*, sXBP1*, XBP1u**	Cox-2*, MPO*	Teng et al. (2017)
Rat	p-PERK*, p-IRE1α*, ATF6f*, XBP1s*, CHOP*, GRP78*	MPO*	Pritchard et al. (2022)

*Up; **down; ERS, endoplasmic reticulum stress; p-, phosphorylation; PERK, PKR-like ER, kinase; eIF2, eukaryotic translation initiation factor 2; ATF4, activating transcription factor 4; CHOP, C/EBP-homologous protein; BiP, binding immunoglobulin protein; IRE1, inositol requiring enzyme 1; ATF6, activating transcription factor 6; XBP1, X-box binding protein 1; GADD153, DNA, damage inducible gene 153; JNK, c-Jun N-terminal kinase; cATF6, cleaved form of ATF6; XBP1s, spliced XBP1; XBP1u, unspliced XBP1; ATF6f, N-terminal fragment of ATF6; GRP78, glucose-regulated protein 78; TNF-α, Tumor Necrosis Factor-α; IL-6, Interleukin-6; IL-1β, interleukin-1β; COX, cyclooxygenase; MPO, myeloperoxidase.

#### 4.2.2 Cellular senescence

Investigations into the epigenetic and transcriptomic profiles of preterm infants at birth, as well as at 14 and 28 days postnatally, have demonstrated increased expression of genes associated with cellular senescence in BPD patients (Cho et al., 2023). Furthermore, primary human fetal lung fibroblasts, when induced to senescence under conditions of moderate hyperoxia (40% oxygen concentration), produce a SASP that exhibits pro-fibrotic effects, which may contribute to impaired lung repair following hyperoxic exposure (You et al., 2019). The study conducted by Jing X et al. provides additional evidence that cellular senescence is a critical factor in the development of BPD following initial induction by hyperoxia (Jing et al., 2024). The research also suggests that the application of tauroursodeoxycholic acid to mitigate ERS presents a promising therapeutic approach to arrest the progression of BPD. Nonetheless, further investigation is required to elucidate the interactions between ERS and UPR pathways in relation to cellular senescence in BPD.

Notably, accumulating evidence extends beyond mere observations of irreversible cell-cycle arrest in senescent lung cells, underscoring their active role in disease progression via the SASP. Specifically, senescent type II alveolar epithelial (AT2) cells and lung fibroblasts secrete pro-inflammatory cytokines (including IL-6 and IL-8), chemokines, and matrix-remodeling enzymes—exerting paracrine effects that sustain inflammation and disrupt the extracellular matrix microenvironment (Coppe et al., 2010). In the immature lung exposed to hyperoxia, persistent SASP signaling has been linked to chronic low-grade inflammation, impaired secondary septation, and alveolar simplification—key pathological hallmarks of the “new” BPD phenotype (Londhe et al., 2011). Reinforcing this causal link, recent *in vivo* studies in neonatal hyperoxia models have shown that targeting senescence-associated pathways alleviates inflammatory injury and improves alveolar development, providing strong *in vivo* evidence that SASP-driven inflammation contributes to BPD pathogenesis (Yao et al., 2023).

#### 4.2.3 Autophagy

Autophagy plays a significant role in lung development and morphogenesis, and hyperoxia-induced BPD has been shown to impair autophagic processes in the lungs of neonatal rats and baboons (Zhang L. et al., 2020). The conditional knockout of the beclin-1 gene (Becn1) in embryonic mouse lung epithelial cells leads to fatal respiratory distress at birth or shortly thereafter, thereby affirming the necessity of intrinsic autophagy for normal lung development (Yeganeh et al., 2019). In mice with BPD, elevated protein levels of LC3B-II, p62, and Lamp1 suggest impaired autophagic processes, and the application of autophagy inducers has been shown to enhance alveolar development and decrease apoptosis (Zhang D. et al., 2018). Comparable outcomes are observed with the use of the autophagy-promoting peptide Tat-P (Zhou et al., 2023), indicating that the activation of autophagic activity can mitigate the pro-inflammatory effects exerted by lung macrophages, thereby protecting the lungs of BPD mice (Soni et al., 2023). Research indicates that BPD mouse models demonstrate notable ER dilation at 14 days postnatal, accompanied by increased autophagy and elevated protein levels of ATF4, CHOP, and LC3B (Li et al., 2018). These findings suggest potential interactions between ERS and autophagy-related pathways in BPD; however, further investigation is required to fully understand their interconnections.

#### 4.2.4 Apoptosis

Exposure to hyperoxia enhances the production of ROS, thereby aggravating changes in the intracellular redox state and inducing oxidative stress. Hyperoxia is also capable of activating ERS-related apoptotic pathway proteins, including CHOP, caspase-12, and caspase-3, which ultimately lead to apoptosis (Xu et al., 2009; Huang et al., 2024).

Furthermore, UPR can lead to increased expression of antioxidants, notably nuclear factor erythroid 2-related factor 2 (Nrf2), a critical transcription factor involved in managing oxidative stress. Nrf2 orchestrates the expression of various antioxidant and phase II detoxification enzyme genes, and is subject to negative regulation by Kelch-like ECH-associated protein 1 (Keap1) (Ngo and Duennwald, 2022). Research indicates that Nrf2 can directly modulate apoptosis by binding to the antioxidant response element (ARE) located between positions −608 and −600, thereby inducing Bcl-xL gene expression, as well as by interacting with the ARER3 region of the Bcl2 gene promoter to increase Bcl2 transcription after Nrf2 translocation to the nucleus (Ma et al., 2022). Nrf2 functions as a central regulator orchestrating the cellular response to the accumulation of misfolded proteins, thereby facilitating a coordinated transcriptional response (He et al., 2020b). Excessive ROS produced by ERS can directly activate Nrf2. Additionally, ROS can lead to the phosphorylation of Nrf2 via stress-induced PERK activation and promote Nrf2 activation through the dissociation of the Nrf2/Keap1 complex (Wang and Kaufman, 2012; Marta Pajares and Rojo, 2017). Concurrently, the activation of Nrf2 can stimulate the expression of genes related to UPR (He et al., 2020a), thereby maintaining intracellular redox homeostasis and inhibiting apoptosis.

Fu R et al. conducted an analysis of blood samples obtained from the umbilical or radial arteries of preterm infants and identified that diminished perinatal cellular glutathione peroxidase (Gpx) activity may increase the risk of developing BPD (Fu et al., 2008). Subsequently, McGrath-Morrow S et al. were the first to elucidate the protective role of Nrf2 and its downstream enzymes, quinone NADH dehydrogenase 1 (NQO1) and Gpx2, in safeguarding alveolar type II cells and promoting alveolar development under conditions of hyperoxic stress-induced oxidative stress in experimental models of BPD (McGrath-Morrow et al., 2009). Zhang et al. demonstrated that glutamine supplementation attenuates lung inflammation, oxidative stress, and apoptosis, while enhancing lung function in a mouse model of hyperoxia-induced BPD, potentially through the inhibition of the IRE1α/JNK signaling pathway (Zhang S. et al., 2023). These results suggest an association between Nrf2 and ERS-induced apoptosis in BPD; however, further investigation is required to elucidate the underlying mechanisms.

### 4.3 The impact of the three UPR branches on BPD pathogenesis

**FIGURE 3:**
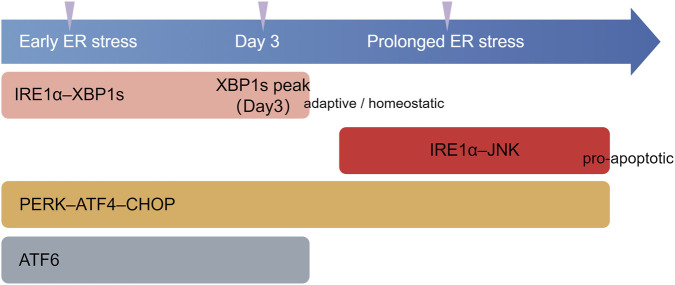
Temporal dynamics of UPR branch activation in hyperoxia-induced BPD. Schematic timeline illustrating the differential temporal activation of unfolded protein response (UPR) branches under hyperoxic conditions. IRE1α is rapidly activated during early endoplasmic reticulum (ER) stress, with spliced XBP1 (XBP1s) reaching a peak around day 3, consistent with an adaptive or homeostatic response. Under prolonged ER stress, XBP1s signaling is attenuated and IRE1α activity shifts toward JNK-associated pro-apoptotic signaling. PERK–ATF4–CHOP signaling is activated throughout hyperoxic exposure and is associated with apoptosis-related responses, whereas ATF6 activation has been reported mainly at early stages, with downstream pathways remaining incompletely defined. This timeline is schematic and reflects relative temporal trends rather than quantitative activation kinetics. ER, endoplasmic reticulum; IRE1, inositol-requiring enzyme 1; IRE1α, inositol-requiring enzyme 1 alpha; XBP1s, spliced X-box binding protein 1; JNK, c-Jun N-terminal kinase; PERK, PKR-like endoplasmic reticulum kinase; ATF4, activating transcription factor 4; CHOP, C/EBP homologous protein; ATF6, activating transcription factor 6.

Current researches corroborate the activation of all three branches of UPR in hyperoxia-induced BPD. Specifically, hyperoxia triggers the PERK/eIF2α/ATF4/CHOP pathway, leading to the upregulation of pro-apoptotic factors such as caspase-3 and Bax, alongside the downregulation of the anti-apoptotic factor BCL (Cui H. et al., 2022). The knockout of CHOP reverses these effects (Choo-Wing et al., 2013; Lu et al., 2017). In models of hyperoxia-induced BPD, IRE1α is rapidly activated following ERS, with downstream XBP1s mRNA reaching its peak on the third day. However, prolonged ERS suppresses XBP1, thereby activating the IRE1α/JNK pathway and inducing apoptosis (Tong et al., 2020). ATF6 is also activated in hyperoxia-induced BPD (Teng et al., 2017), yet further investigation is required to elucidate the ATF6-mediated signaling pathways involved in the pathogenesis of BPD (Figure 3).

Studies indicate that the ubiquitin-proteasome pathway (UPP) may play a critical role in the ERS-mediated apoptosis of alveolar type II epithelial cells induced by hyperoxia, thereby contributing to BPD development (Zhu et al., 2021). Research conducted by Choo-Wing R et al. demonstrates that COX2, functioning as an upstream mediator of the UPR, is upregulated under conditions of hyperoxia, thereby facilitating the initiation of the UPR and contributing to lung damage and inflammation (Choo-Wing et al., 2013). Furthermore, Pritchard et al. suggest that the accumulation of oxidative stress induced by myeloperoxidase (MPO) is a prerequisite for ERS, with prolonged ERS playing a pivotal role in neonatal lung injury and the development of BPD (Pritchard et al., 2022).

In conclusion, the three branches of the UPR are critically involved in various pathogenic mechanisms of BPD. However, further comprehensive and detailed investigations are necessary to elucidate their specific roles and underlying mechanisms.

### 4.4 Cell type–specific roles of ERS/UPR in BPD

The impact of endoplasmic reticulum stress (ERS) and the unfolded protein response (UPR) in bronchopulmonary dysplasia is increasingly understood to be cell-specific, with distinct and often divergent roles across lung cell types. In AT2 epithelial cells, hyperoxia potently triggers ERS and activates pro-apoptotic UPR signaling—particularly through the PERK–eIF2α–ATF4 axis and IRE1α pathway—leading to elevated CHOP expression, AT2 cell loss, and impaired surfactant production and alveolarization (Lu et al., 2015; Pritchard et al., 2022; Wu et al., 2024). Interestingly, transient ATF4 activation may initially support AT2 cell proliferation and metabolic adaptation, indicating that the UPR can shift from an adaptive to a deleterious response depending on the duration and intensity of stress (Yee et al., 2022).

In the pulmonary endothelium, ERS contributes to the disruption of angiogenic signaling and capillary network formation, a key feature of the vascular phenotype observed in BPD (Obst et al., 2022; Pritchard et al., 2022; Zanini et al., 2023). Lung fibroblasts, when exposed to moderate hyperoxia, often undergo premature senescence accompanied by a profibrotic secretory phenotype (SASP), which can disrupt normal extracellular matrix remodeling and alveolar septation (You et al., 2019). Similarly, hyperoxia induces senescence and metabolic shifts in lung epithelial cells, with the timing and cellular context of senescence—particularly in AT2 cells and secondary crest myofibroblasts—critically influencing postnatal lung development and injury patterns (Scaffa et al., 2021; Yao et al., 2023).

Within immune cells such as alveolar macrophages and neutrophils, ERS/UPR signaling interfaces with inflammatory pathways including NF-κB, amplifying cytokine production and sustaining lung inflammation. Experimental inhibition of ER stress in injury models reduces NF-κB activation and inflammatory mediator release, highlighting ERS as an upstream regulator of immune-mediated damage (Desmarquest et al., 1998; Bhandari, 2010; Kim et al., 2013; Barabutis, 2020).

Collectively, these findings illustrate how ERS/UPR activation differentially directs repair and injury across AT2 cells, endothelial cells, fibroblasts, and immune cells in the developing lung. A clearer understanding of the cell-type-specific roles of UPR signaling in the developing lung will require the integration of high-resolution cell mapping with functionally targeted experimental systems. Recent single-cell studies in neonatal hyperoxia models have shown that epithelial, endothelial, mesenchymal, and immune compartments do not respond uniformly to injury, but instead follow distinct transcriptional and intercellular communication trajectories, supporting the view that UPR activation should be interpreted within defined cellular contexts rather than as a bulk tissue response (Hurskainen et al., 2021; Cantu et al., 2023). Spatial transcriptomic analysis of human infant lungs further suggests that developmental stage, tissue architecture, and local cellular neighborhoods need to be considered together when assessing stress-responsive signaling pathways (Mallapragada et al., 2025). These descriptive frameworks could then be coupled with lineage-tracing and cell-restricted genetic strategies to determine whether PERK, IRE1α/XBP1s, ATF6, or CHOP exert adaptive or maladaptive effects in specific epithelial, endothelial, mesenchymal, or immune populations (Sun et al., 2022; Meng et al., 2023). Complementary *ex vivo* platforms, including lung organoids and precision-cut lung slices, may further help resolve multicellular interactions and niche-dependent responses that are difficult to dissect in conventional monoculture systems (Vazquez-Armendariz and Tata, 2023; Lehmann et al., 2025; Zanetto et al., 2025). Together, these approaches may clarify not only which cell populations are most vulnerable to ER stress during lung development, but also when and where UPR signaling shifts from an adaptive program to a driver of arrested alveolar and vascular development.

### 4.5 Current controversies and unresolved mechanisms in ERS/UPR-mediated BPD

While considerable advances have been made in characterizing ER stress and UPR signaling in hyperoxic lung injury, several key mechanistic questions remain actively debated. A central unresolved issue is whether the activation of the UPR in the premature lung serves a fundamentally adaptive or injurious role. In neonatal models, early-phase hyperoxia can induce moderate UPR signaling alongside antioxidant responses, which may help to mitigate initial oxidative damage. The observation that chemical chaperones like tauroursodeoxycholic acid can improve alveolarization under these conditions further suggests that a regulated UPR can be protective (Pritchard et al., 2022). However, under conditions of prolonged or severe ER stress, the UPR appears to pivot toward promoting apoptosis. For instance, sustained induction of the pro-apoptotic factor CHOP correlates with increased epithelial cell death and arrested alveolar development in neonatal models (Lu et al., 2015). The contrasting finding that CHOP can limit injury in adult hyperoxia models underscores the profound developmental and contextual specificity of UPR outcomes (Lozon et al., 2011). A critical gap in knowledge is the precise point at which this protective-to-detrimental switch occurs in the developing lung.

The role of autophagy in BPD pathogenesis remains debated. Some studies report that hyperoxia increases autophagy markers, and that enhancing autophagic activity—via mTOR inhibition or RPTOR suppression—reduces apoptosis and preserves alveolar structure, supporting a cytoprotective role (Sureshbabu et al., 2016). Other evidence, however, indicates that autophagic flux becomes impaired during injury progression. In neonatal animals, hyperoxia causes p62 accumulation together with reduced AMPK activation, and haploinsufficiency of Becn1 further aggravates alveolar damage, suggesting that insufficient or stalled autophagy contributes to worse outcomes (Zhang L. et al., 2020). Mechanistically, the early downregulation of Syntaxin 17 (STX17) in hyperoxic rat lungs disrupts autophagosome-lysosome fusion; interventions that restore this flux improve epithelial cell survival (Zhang D. et al., 2020). Determining whether the predominant pathophysiology involves autophagy failure or compensatory hyperactivation likely depends on disease stage and specific cellular context.

Further complexity arises from the dual signaling outputs of IRE1α. Its canonical role in splicing Xbp1 to generate the transcription factor XBP1s enhances ER homeostasis and is generally cytoprotective. Under chronic or overwhelming stress, however, IRE1α can activate the Regulated IRE1-Dependent Decay (RIDD) pathway, which degrades RNAs and promotes JNK-mediated apoptosis. How this critical balance between adaptive (XBP1s) and pro-death (RIDD) signaling is maintained in the premature lung is unknown. Activated IRE1α is present in alveolar cells during hyperoxia, but whether it primarily drives adaptation or contributes to injury remains unclear (Siwecka et al., 2021).

These ongoing controversies underscore significant gaps in our understanding of how ER stress, UPR signaling, and autophagy are integrated within the unique developmental milieu of the premature lung. Resolving these mechanistic uncertainties is essential for refining therapeutic strategies that aim to bolster adaptive cellular stress responses while inhibiting maladaptive pathways that drive injury.

## 5 Therapeutic modulation of ER stress and UPR pathways in BPD: evidence, mechanistic basis, and translational readiness

Despite extensive preclinical evidence implicating ER stress (ERS) and the unfolded protein response (UPR) in hyperoxia-induced lung injury, translation of UPR-modulating therapies to clinical practice remains limited. This section provides a rigorously evidence-based synthesis of the major therapeutic candidates relevant to ERS/UPR biology in bronchopulmonary dysplasia (BPD), explicitly distinguishing established human clinical evidence, preclinical mechanistic support, and speculative interpretations.

### 5.1 IGF-1/IGFBP-3

#### 5.1.1 Human clinical evidence (FACT)

A multicenter phase II randomized trial (NCT01096784) evaluated recombinant hIGF-1/hIGFBP-3 in extremely preterm infants. The intervention did not significantly reduce retinopathy of prematurity, although a nonsignificant trend toward lower severe BPD was reported (Ley et al., 2019). In addition, a longitudinal cohort study of 108 very preterm infants showed that lower postnatal serum IGF-1 concentrations during the first weeks of life were independently associated with later development of BPD, after adjustment for gestational age and sex (Lofqvist et al., 2012).

#### 5.1.2 Mechanistic evidence

In hyperoxia-exposed neonatal rodents, IGF-1 reduces PERK/eIF2α/ATF4/CHOP activation, oxidative stress, and apoptosis, improving alveolar development (Cui H. et al., 2022).

#### 5.1.3 Interpretation

Although biologically plausible, no human studies have demonstrated direct modulation of UPR signaling by IGF-1, and its potential benefit for BPD remains hypothesis-generating.

### 5.2 Caffeine

#### 5.2.1 Human clinical evidence (FACT)

The landmark CAP Trial (NCT00182312) demonstrated that caffeine significantly reduces BPD incidence and shortens ventilatory support in very low birth weight infants (Schmidt et al., 2006). In the 11-year follow-up of the trial (Schmidt et al., 2017), neonatal caffeine therapy did not increase the overall risk of long-term neurodevelopmental impairment and was associated with a lower rate of motor impairment, supporting the sustained safety of standard-dose caffeine into school age.

#### 5.2.2 Mechanistic evidence

Experimental studies in hyperoxia-exposed neonatal rodents suggest that caffeine may act as a chemical chaperone, attenuating ER stress by reducing GRP78/BiP and CHOP expression and thereby ameliorating oxidative lung injury (Teng et al., 2017).

#### 5.2.3 Interpretation

Caffeine is the only therapy with robust RCT evidence of reducing BPD, though its UPR-modulating role in humans remains inferred rather than proven.

### 5.3 Vitamin A

#### 5.3.1 Human clinical evidence (FACT)

Vitamin A supplementation for prevention of BPD has been evaluated in a series of trials conducted by the NICHD Neonatal Research Network. An early randomized study raised the question of whether the dose used was sufficient to achieve a clear clinical effect (Kennedy et al., 1997). A subsequent large, well-designed randomized controlled trial (NCT01203488) demonstrated that intramuscular vitamin A produced a modest but statistically significant reduction in the composite outcome of BPD or death in extremely low birth weight infants (Tyson et al., 1999). Follow-up at 18–22 months showed no major adverse neurodevelopmental signal, while the modest short-term pulmonary benefit was maintained at the population level (Ambalavanan et al., 2005).

#### 5.3.2 Mechanistic evidence

Vitamin A and its active metabolite all-trans retinoic acid play essential roles in lung development, including airway and alveolar epithelial differentiation, alveolar septation, and surfactant synthesis (Timoneda et al., 2018; Fernandes-Silva et al., 2020). Experimental studies in non-pulmonary injury models further show that retinoic acid can modulate cellular stress responses—enhancing antioxidant defenses and attenuating ER-stress-related apoptotic signaling, such as by reducing CHOP and caspase-12 activation (Zhou et al., 2016). However, direct evidence of ER-stress modulation by vitamin A in neonatal lung or BPD models is currently lacking.

#### 5.3.3 Interpretation

Although retinoic acid can modulate ER-stress pathways in other injury models, vitamin A has not been shown to directly influence ER-stress or UPR signaling in neonatal lung or BPD. Thus, its modest clinical benefit in BPD is most plausibly attributed to its developmental and maturational effects on the immature lung, rather than to a direct UPR-modulating mechanism.

### 5.4 Glutamine

#### 5.4.1 Human clinical evidence (FACT)

Glutamine supplementation has been tested in multiple randomized controlled trials using both parenteral and enteral routes in preterm and very-low-birth-weight infants. A large multicenter trial of parenteral glutamine conducted by the NICHD Neonatal Research Network (NCT00005775) found no reduction in death, late-onset sepsis, or other major short-term morbidities, including BPD, in extremely low birth weight infants (Poindexter et al., 2004).Smaller enteral trials reported inconsistent benefits for late-onset sepsis or feeding tolerance—some suggesting reduced infectious morbidity (Neu et al., 1997; Vaughn et al., 2003; van den Berg et al., 2005), but none demonstrated a significant decrease in BPD incidence. A Cochrane review pooling available RCTs concluded that routine glutamine supplementation does not reduce mortality or major neonatal morbidities, including BPD (Moe-Byrne et al., 2016).

#### 5.4.2 Mechanistic evidence

Glutamine may protect the pulmonary function of neonatal rats against hyperoxia-induced damage by inhibiting ERS through the IRE1α/JNK pathway, enhancing alveolar development, and decreasing the production of pro-inflammatory cytokines and inflammatory cells (Zhang S. et al., 2023).

#### 5.4.3 Interpretation

Current clinical data do not support glutamine as an effective strategy for preventing BPD in preterm infants, and no trial has been designed to directly target ER-stress or UPR endpoints. Its potential UPR-modulating and lung-protective effects remain theoretical, grounded in preclinical models rather than demonstrated clinical benefit.

### 5.5 TUDCA

#### 5.5.1 Human clinical evidence (FACT)

Tauroursodeoxycholic acid (TUDCA) has not been evaluated in preterm infants or in any clinical trial targeting BPD. No neonatal pharmacokinetic or dosing studies are available, and no BPD-related trials are registered on ClinicalTrials.gov. Existing human data come mainly from adult hepatobiliary disorders, where TUDCA has demonstrated safety and biochemical efficacy.

#### 5.5.2 Mechanistic evidence

In neonatal hyperoxia- or tunicamycin-induced lung injury models, TUDCA functions as a chemical chaperone that reduces ER stress markers (GRP78, XBP1s, CHOP), restores epithelial cell homeostasis, decreases inflammatory cell infiltration, and improves alveolar development (Pritchard et al., 2022). Complementary reviews highlight TUDCA as one of the most consistent ER-stress–modulating agents in experimental BPD models (Wu et al., 2024).

#### 5.5.3 Interpretation

Although mechanistically promising, TUDCA lacks any neonatal clinical evidence relevant to BPD, and essential translational information—including pharmacokinetics, safety, and appropriate dosing in preterm infants—remains unknown. Its potential benefit in BPD therefore remains entirely preclinical and hypothesis-generating.

### 5.6 Other preclinical ER-stress–modulating agents

#### 5.6.1 IFNγ pathway modulation

Genetic deletion of interferon-γ reduces COX-2 induction and lowers BiP and CHOP expression in hyperoxia-induced BPD mouse models, attenuating epithelial injury and apoptosis (Choo-Wing et al., 2013).

#### 5.6.2 N-acetyl-lysyl-tyrosyl-cysteine amide (KYC)

KYC reduces myeloperoxidase (MPO)–driven ER stress, inflammation, and apoptosis in neonatal hyperoxia models, improving lung architecture (Jing et al., 2024).

#### 5.6.3 Proteasome inhibitor MG132

MG132 exacerbates ER stress by increasing GRP78, PERK, ATF4, ATF6, and CHOP expression in alveolar type II epithelial cells, leading to enhanced apoptosis under hyperoxia or tunicamycin exposure (Zhu et al., 2021).

#### 5.6.4 Calcitonin gene-related peptide (CGRP)

CGRP attenuates hyperoxia-induced apoptosis, oxidative stress, and ROS generation in type II alveolar epithelial cells. Its protective effect may involve Notch1/HERP signaling and enhanced ER-associated degradation (ERAD) (Bai et al., 2017).

#### 5.6.5 ERp57 modulation

Overexpression of the ER chaperone ERp57 intensifies ER stress and apoptosis in endothelial cells under hyperoxia or tunicamycin challenge, whereas ERp57 knockdown is protective through reduced caspase-3 activation and enhanced BiP/GRP78 induction (Xu et al., 2009).

## 6 Conclusion

BPD remains a major challenge in neonatal medicine, arising from the interplay of inflammatory, oxidative, and developmental disturbances that collectively impair alveolar and vascular maturation in extremely preterm infants. Increasing evidence indicates that ER stress and the UPR interact with several pathological processes central to BPD—including inflammation, autophagy, senescence, and apoptosis—yet the precise contribution of UPR signaling to disease onset and progression remains incompletely understood. The dual adaptive–injurious nature of UPR activation, its diverse upstream triggers, and the cell-type–specific responses of alveolar epithelial, endothelial, mesenchymal, and immune cells further complicate attempts to define its role *in vivo*.

Although multiple interventions modulate ER stress pathways in experimental models, translation into clinical practice remains limited. Caffeine continues to be the only therapy with reproducible reductions in BPD, and vitamin A offers a modest benefit in the composite outcome of BPD or death. Importantly, neither therapy has been shown to exert its effects through confirmed modulation of UPR signaling in human infants. In contrast, agents with strong UPR-targeting activity—such as TUDCA or KYC—remain confined to preclinical studies, underscoring a persistent divide between mechanistic insights and therapeutic application. As summarized in Table 2, this divergence between experimental efficacy and clinical readiness remains one of the major translational barriers in the development of ER-targeted therapies for BPD.

**TABLE 2:** Summary of therapeutic and experimental agents influencing ER stress and UPR pathways in bronchopulmonary dysplasia.

Intervention	Evidence level	Human clinical evidence	ERS/UPR mechanistic evidence	Translational interpretation
IGF-1/IGFBP-3	Phase II RCT + observational	NCT01096784; no significant ↓ROP; nonsignificant ↓severe BPD; low IGF-1 associated with ↑BPD risk	↓PERK/eIF2α/ATF4/CHOP; ↓oxidative stress; ↑alveolarization	Plausible mechanistically; no human UPR evidence
Caffeine	Large RCT + long-term follow-up	NCT00182312; ↓BPD; sustained safety	↓GRP78/BiP, ↓CHOP; chemical-chaperone-like effects	Only therapy with proven BPD benefit; UPR impact inferred
Vitamin A (Retinol)	Multiple RCTs	NCT01203488; modest ↓BPD/death; safe in follow-up	Supports differentiation; RA reduces CHOP/Caspase-12 in non-pulmonary models	Likely acts via lung maturation, not UPR
Glutamine	Multiple RCTs + Cochrane review	No ↓BPD; inconsistent ↓sepsis	Inhibits IRE1α-JNK; ↓inflammation; ↑alveolar structure	Not effective clinically; UPR effects preclinical
TUDCA	Preclinical only	—	↓GRP78, XBP1s, CHOP; restores epithelial homeostasis; ↑alveolarization	Mechanistically strong; no neonatal safety data
IFNγ pathway modulation	Preclinical only	—	IFNγ deletion ↓COX-2, BiP, CHOP; ↓epithelial apoptosis	Highlights inflammatory–ERS crosstalk; no translational data
KYC	Preclinical only	—	↓MPO-driven ER stress; ↓apoptosis; ↑alveolarization	Early-stage ERS inhibitor; no human evidence
MG132 (proteasome inhibitor)	Preclinical only	—	Proteasome inhibition ↑GRP78, PERK, ATF4, ATF6, CHOP; ↑apoptosis	Experimental tool; not a therapeutic candidate
CGRP	Preclinical only	—	↓Apoptosis/oxidative stress; may enhance ERAD via Notch1/HERP	Protective in models; no translational evidence
ERp57 modulation	Preclinical only	—	ERp57 overexpression ↑ERS/apoptosis; knockdown ↑BiP/GRP78	Demonstrates ER chaperone sensitivity; not therapeutic

ERS, endoplasmic reticulum stress; UPR, unfolded protein response; RCT, randomized controlled trial; BPD, bronchopulmonary dysplasia; AECII, alveolar type II, epithelial cells; IGF-1, insulin-like growth factor-1; IGFBP-3, insulin-like growth factor binding protein-3; TUDCA, tauroursodeoxycholic acid; KYC, N-acetyl-lysyl-tyrosyl-cysteine amide; CGRP, calcitonin gene-related peptide; RA, all-trans retinoic acid; ERAD, endoplasmic reticulum–associated degradation; PK, pharmacokinetics; COX-2, cyclooxygenase-2; MPO, myeloperoxidase; BiP (GRP78), binding immunoglobulin protein; CHOP, C/EBP, homologous protein; XBP1s, spliced X-box–binding protein 1; PERK, protein kinase RNA-like ER, kinase; ATF4, activating transcription factor 4; ATF6, activating transcription factor 6; IRE1α, inositol-requiring enzyme 1 alpha; JNK, c-Jun N-terminal kinase.

Symbols: ↑, increase; ↓, decrease.

“—” indicates absence of neonatal or BPD-specific clinical trials.

“Mechanistic evidence” summarizes experimental observations on ERS/UPR-related pathways derived primarily from preclinical studies and does not imply proven clinical efficacy.

Closing this gap will require early-phase clinical studies that integrate mechanistic biomarkers to assess target engagement and biological effect. Measuring ER stress markers (e.g., BiP/GRP78, CHOP, XBP1s, ATF4) in accessible biospecimens, together with stratification by oxygen exposure, inflammatory burden, and developmental stage, will be essential for identifying infants most likely to benefit. Attention to therapeutic timing is equally important, as UPR activation transitions from adaptive to maladaptive phases during injury and repair—defining the window during which intervention is likely to be effective.

As understanding of ER stress–driven lung injury continues to advance, the prospect of developing rational, UPR-based therapies for BPD becomes increasingly tangible. Establishing a more robust interface between basic discovery and translational trial design will be crucial for determining whether targeted modulation of ER stress can meaningfully alter lung development and long-term outcomes in vulnerable preterm infants.
